# Survival Outcomes After Breast-Conserving Therapy Compared With Mastectomy for Patients With Early-Stage Invasive Micropapillary Carcinoma of the Breast: A SEER Population-Based Study

**DOI:** 10.3389/fonc.2021.741737

**Published:** 2021-11-01

**Authors:** Song Wang, Yiyuan Zhang, Fangxu Yin, Xiaohong Wang, Zhenlin Yang

**Affiliations:** ^1^ Department of Thyroid and Breast Surgery, Binzhou Medical University Hospital, Binzhou, China; ^2^ Department of Reproductive Endocrinology, Affiliated Reproductive Hospital of Shandong University, Jinan, China

**Keywords:** invasive micropapillary carcinoma, SEER, mastectomy, BCT, survival

## Abstract

**Background:**

Invasive micropapillary breast carcinoma (IMPC) is a relatively rare pathological type of invasive breast cancer. Little is currently known on the efficacy and safety of breast-conserving treatment (BCT, lumpectomy plus postsurgical radiation) compared with mastectomy in women diagnosed with early-stage IMPC. Accordingly, we sought to investigate the long-term prognostic differences between BCT and mastectomy in patients with T1-3N0-3M0 invasive micropapillary breast carcinoma using data from the Surveillance, Epidemiology, and End Results (SEER) database.

**Materials and Methods:**

We retrospectively analyzed 1,203 female patients diagnosed with early-stage IMPC between 2004 and 2015 from the SEER database. The impact of different surgical approaches on patient prognosis was assessed by the Kaplan-Meier method and Cox proportional risk models.

**Results:**

A total of 609 and 594 patients underwent mastectomy and BCT, respectively. Compared with patients who underwent a mastectomy, patients in the BCT group were older and had lower tumor diameters, lower rates of lymph nodes metastasis, and higher rates of ER receptor positivity and PR receptor positivity (*p* < 0.05). Kaplan-Meier plots showed that the overall survival (OS) and breast cancer-specific survival (BCSS) were higher in the BCT group than in the mastectomy group. In subgroup analysis, patients with T2 stage in the BCT group had better OS than the mastectomy group. Multivariate analysis showed no statistical difference in OS and BCSS for patients in the mastectomy group compared with the BCT group (hazard ratio (HR) = 0.727; 95% confidence interval (95% CI) 0.369–1.432, *p* = 0.357; HR = 0.762; 95% CI 0.302–1.923, *p* = 0.565; respectively). During the multivariate analysis and stratifying for the T stage, a better OS was found for patients with T2 stage in the BCT group than the mastectomy group (HR = 0.333, 95% CI: 0.149–0.741, *p* = 0.007). There was no significant difference in OS for patients with T1 and T3 stages between the BCT and mastectomy groups (*p* > 0.05).

**Conclusion:**

In women with early-stage IMPC, BCT was at least equivalent to mastectomy in terms of survival outcomes. When both procedures are feasible, BCT should be recommended as the standard surgical treatment, especially for patients with T2 disease.

## Introduction

Invasive micropapillary carcinoma (IMPC) is a rare subtype of breast cancer (1). According to the current literature, it accounts for approximately 3%–6% of all breast cancers (2, 3). Fisher et al. (4) first introduced the concept of “micropapillary structures” in breast tumors in 1980, when they observed a “mulberry-like appearance” under electron microscopy. In contrast, the definition of IMPC was first established by Siriaunkgu and Tavassoli in 1993 (5). IMPC has been characterized with low incidence and high malignancy rates and a marked tendency for lymphatic duct infiltration, regional lymph node metastasis, and local recurrence. IMPC is also widely recognized for its specific morphological structure, aggressive biological behavior, and poor prognosis (3, 6, 7). In the latest WHO (2003) classification of breast tumors, IMPC has been classified as a special type of breast cancer (2). Due to its rarity, the impact of surgical modalities on the prognosis of early-stage IMPC has not been determined. Moreover, there is a paucity of recommendations on the choice of surgical modality for IMPC in clinical guidelines.

Over the past 30 years, several randomized trials (RCTs) on BCT and mastectomy have concluded that these two treatments led to the same prognosis in breast cancer patients (8–10). However, these trials were initiated in the 1970s and 1980s. In recent decades, improvements in screening equipment and instruments, as well as in the systemic treatment of breast cancer and radiation therapy, have improved the detection and survival rates of patients with early-stage breast cancer, which has facilitated the gradual replacement of breast-conserving surgery by wide local excision as a better surgical option (11, 12). Recently, several large sample studies from different countries and regions have shown that BCT had higher survival rates than mastectomy for patients with early-stage breast cancer (13–16). Current clinical guidelines recommend breast-conserving surgery for patients with stages I and II breast cancer when contraindications for breast-conserving surgery are ruled out. After downstaging with neoadjuvant chemotherapy, BCT can also be considered for some patients with stage III disease (17). Nonetheless, the prognosis of IMPC patients undergoing BCT and mastectomy is unclear. The high incidence of lymph node metastasis and lymphovascular invasion associated with IMPC makes it challenging for surgeons who choose BCT.

The present study used the Surveillance, Epidemiology, and End-results (SEER) database of the US National Cancer Institute registry to determine the differences in patient survival for each treatment modality. This database can be used to compare the prognostic differences associated with different treatments for various cancers due to its large sample size and long-term follow-up data. Using the SEER database, we sought to assess the survival differences between BCT and mastectomy in T1-3N0-3M0 IMPC patients.

## Materials and Methods

### Data Sources and Patient Selection

**Figure 1:**
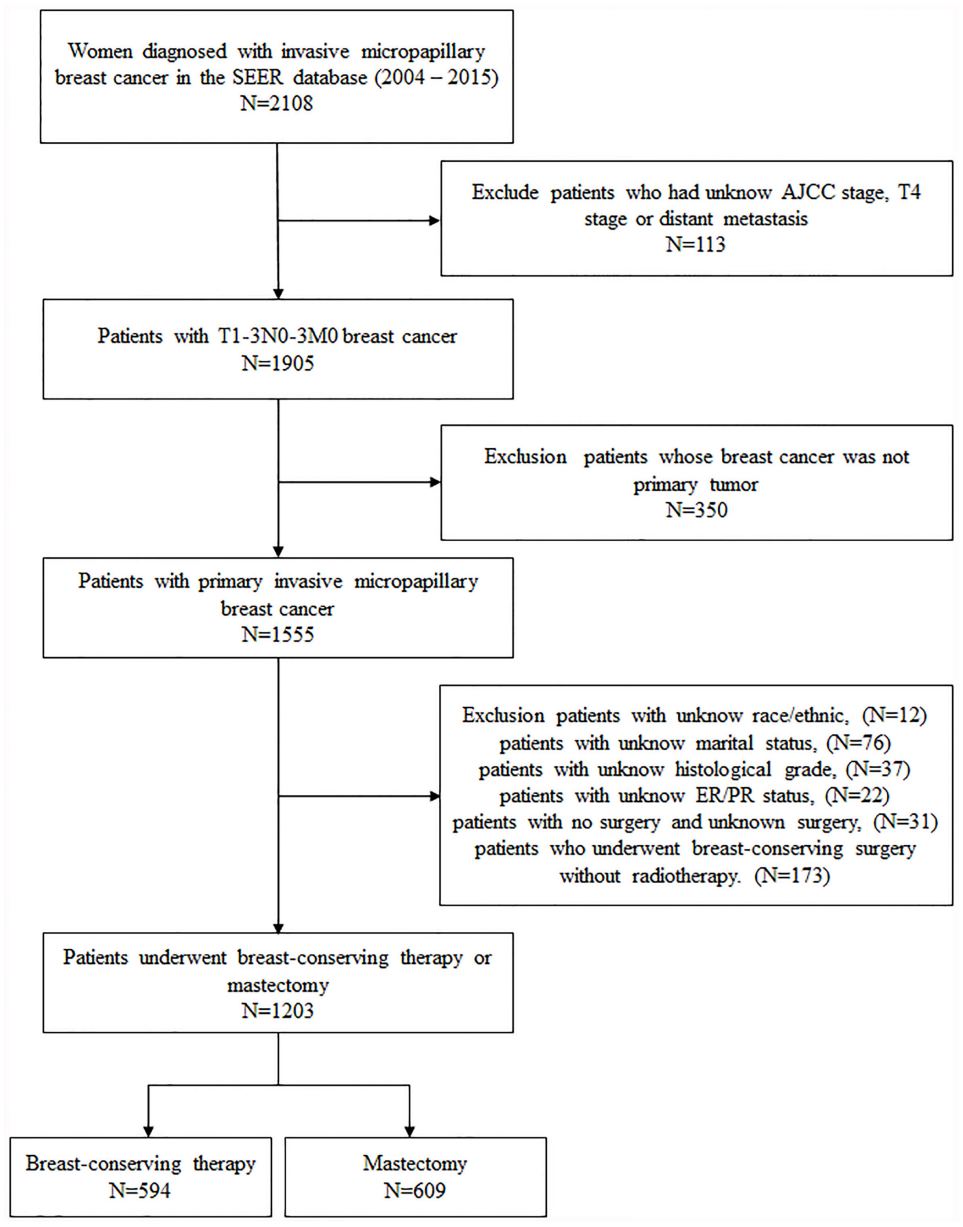
Flow chart of the study cohort.

All patient information, including demographics, diagnosis time, marital status, tumor features, type of surgery, radiotherapy, survival months, and survival status, were obtained from the SEER database. Women diagnosed with unilateral invasive micropapillary carcinoma (ICD-O-3 Code 8507/3) from 2004 to 2015 were selected. The inclusion criteria consisted of breast cancer patients staged T1-3N0-3M0 based on the sixth edition of the American Cancer Commission (AJCC) staging system; breast cancer is the first primary tumor; and patients with complete demographic information and laboratory results for estrogen receptor (ER) and progesterone receptor (PR) positivity. Patients who underwent breast-conserving surgery without radiotherapy were excluded from the study. Finally, 1,203 patients were included in this study. According to the surgical approach, the whole cohort was divided into two groups: breast-conserving therapy (BCT, *n* = 594) and mastectomy (*n* = 609) (**Figure 1**).

### Statistical Analysis

The Pearson chi-square test and Fisher exact probability method were used to compare the characteristics of the BCT and mastectomy groups. The overall survival (OS) and the breast cancer-specific survival (BCSS) of the two groups were analyzed by Kaplan-Meier and log-rank test. In addition, after stratifying for the T and N stages based on the sixth edition of the AJCC staging system, the survival results of the two groups were analyzed and compared. Cox proportional hazards model was used to calculate 95% confidence interval (CI) and hazard ratio (HR) of OS and BCSS. All the tests were bilateral, and *p* < 0.05 was used to denote statistical significance. All analyses were performed using R language software (http://www.R-project.org, The R Foundation).

## Results

### Patient Characteristics

One thousand two hundred three patients with IMPC (T1-3N0-3M0) were analyzed in our study, among which 594 (49.4%) and 609 (50.6%) received BCT and mastectomy, respectively (**Table 1**). The median follow-up time was 57.49 months. Most patients in the BCT group were ≥50 years old (86.2% *vs.* 67.5%), white (78.8% *vs.* 77.0%), and had lower AJCC stage (52.9% *vs.* 23.0%). The BCT group also had a lower proportion of larger-sized tumors (>2 cm) (73.4% *vs.* 42.9%, *p* < 0.001) and low lymph node metastasis rate (64.0% *vs.* 34.2%, *p* < 0.001) and chemotherapy rate (59.3% *vs.* 35.5%, *p* < 0.001) than the mastectomy group. Moreover, a higher percentage of patients with positive ER and PR receptors were found in the BCT group than the mastectomy group (93.3% *vs.* 86.7%, *p* < 0.001; 83.3% *vs.* 75.7%, *p* < 0.001, respectively).

**Table 1:** Comparison of baseline characteristics of early-stage IMPC between BCT and mastectomy groups.

Characteristics	Patients, No. (%)		*p*-value
	BCT	Mastectomy	
**Age (years)**			<0.001
<50	82 (13.8)	198 (32.5)	
50–64	243 (40.9)	226 (37.1)	
65–80	235 (39.6)	157 (25.8)	
>80	34 (5.7)	28 (4.6)	
**Race**			0.595
White	468 (78.8)	469 (77.0)	
Black	63 (10.6)	76 (12.5)	
Others	63 (10.6)	55 (10.5)	
**Marital status**			0.492
Married	352 (59.3)	349 (57.3)	
Single	242 (40.7)	260 (42.7)	
**Grade**			0.016
I	56 (9.4)	45 (7.4)	
II	345 (58.1)	320 (52.5)	
III	189 (31.8)	232 (38.1)	
IV	4 (0.7)	12 (2.0)	
**AJCC stage**			<0.001
I	314 (52.9)	140 (23.0)	
II	235 (39.5)	261 (42.9)	
III	45 (7.6)	208 (34.2)	
**T stage**			<0.001
T1	436 (73.4)	261 (42.9)	
T2	147 (24.7)	251 (41.2)	
T3	11 (1.9)	97 (15.9)	
**N stage**			<0.001
N0	380 (64.0)	208 (34.2)	
N1	175 (29.5)	219 (36.0)	
N2	26 (29.0)	102 (16.7)	
N3	13 (2.2)	80 (13.1)	
**ER status**			<0.001
Negative	40 (6.7)	81 (13.3)	
Positive	554 (93.3)	528 (86.7)	
**PR status**			0.001
Negative	100 (16.8)	148 (24.3)	
Positive	494 (83.2)	461 (75.7)	
**Radiation**			<0.001
No	0 (0.0)	390 (64.0)	
Yes	594 (100.0)	219 (36.0)	
**Chemotherapy**			<0.001
No	352 (59.3)	216 (35.5)	
Yes	242 (40.7)	393 (64.5)	

BCT, breast-conserving therapy.

### Survival Outcomes Between Mastectomy Group and BCT Group in Overall and Subgroup Analysis

**Figure 2:**
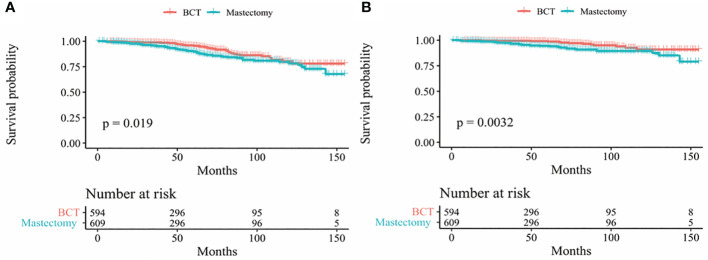
Kaplan-Meier survival curves **(A)** OS between mastectomy and BCT group in the entire cohort; **(B)** BCSS between mastectomy and BCT group in the entire cohort. OS, overall survival; BCSS, breast cancer-specific survival; BCT breast-conserving therapy.

**Figure 3:**
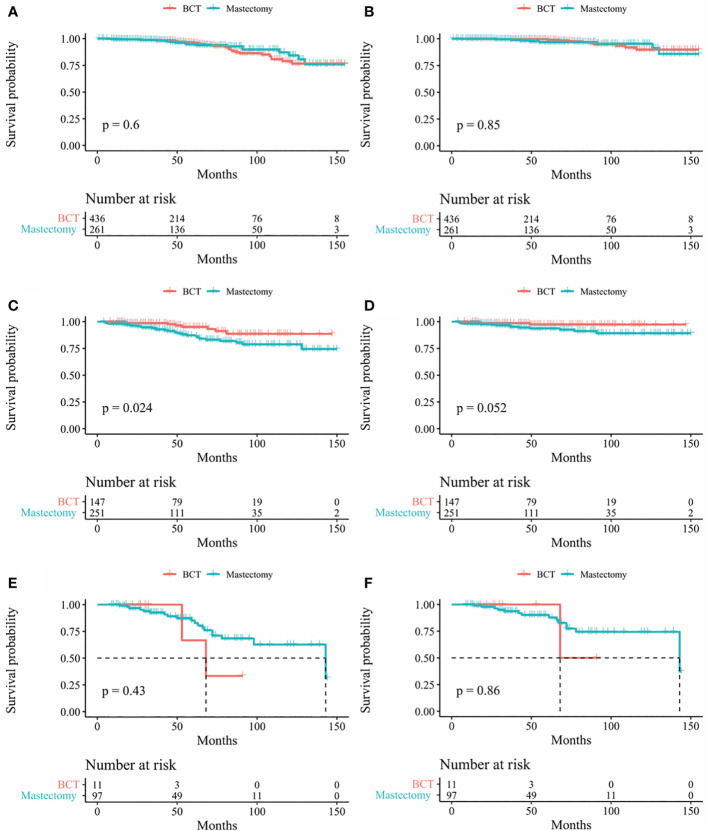
Kaplan-Meier survival curves of subgroup stratified by T stage **(A)** OS between mastectomy group and BCT group in patients with T1 stage **(B)** BCSS between mastectomy group and BCT group in patients T1 stage; **(C)** Os between mastectomy goup and BCT group in patients with T2 stage **(D)** BCSS between mastectomy group and BCT group in patients with T2 stage **(E)** Os between mastectomy group and BCT group in patients with T3 stage; **(F)** BCSS between mastectomy group and BCT group in patients with T3 stage. OS, overall survival; BCSS, breast cancer-specific survival; BCT, breast-conserving therapy.

**Figure 4:**
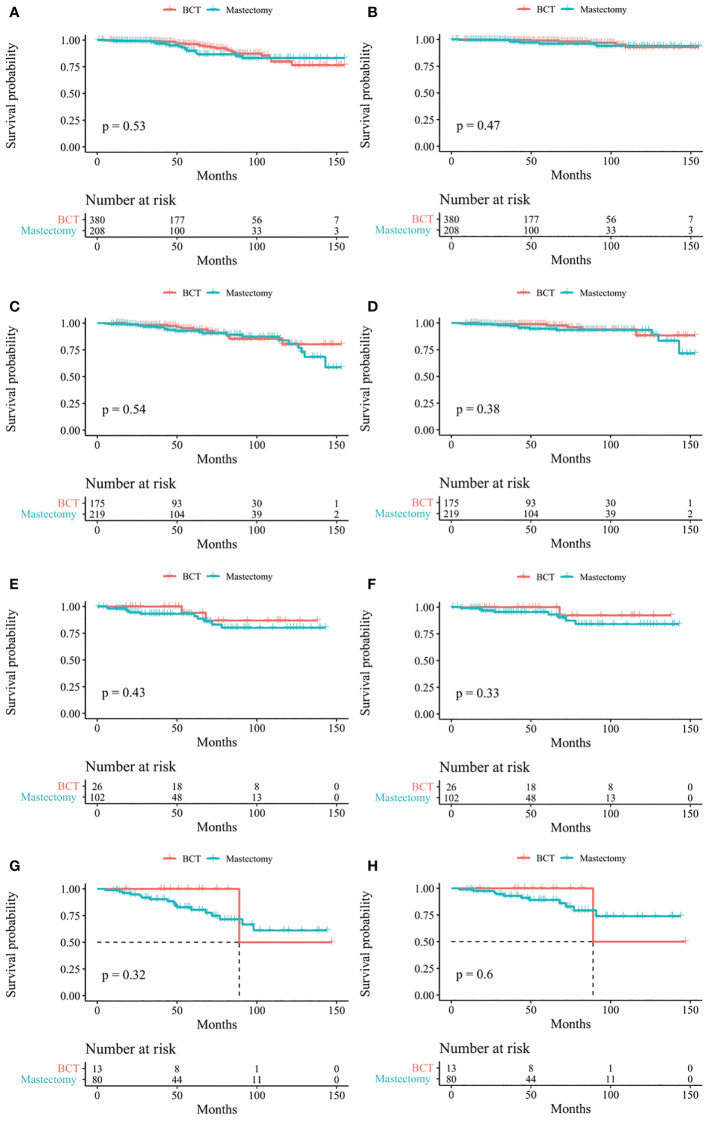
Kaplan-Meier survival curves of subgroup stratified by N stage **(A)** OS between mastectomy group and BCT group in patients with N0 stage **(B)** BCSS between mastectomy group and BCT group in patients with N0 stage **(C)** Os between mastectomy goup and BCT group in patients with N1 stage **(D)** BCSS between mastectomy group and BCT group in patients with N1 stage **(E)** Os between mastectomy group and BCT group in patients with N2 stage **(F)** BCSS between mastectomy group and BCT group in patients with N2 stage **(G)** OS between mastectomy group and BCT group in patients with N3 stage **(H)** BCSS between mastectomy group and BCT group in patients with N3 stage. OS, overall survival; BCSS, breast cancer specific survival; BCT, breast conserving therapy.

In our study, Kaplan-Meier curves were used to access the OS and BCSS of patients in the entire cohort and subgroups of patients stratified for T and N stages. Patients who underwent BCT had significantly improved OS and BCSS (*p* < 0.05) than those who received mastectomy (**Figure 2**). After stratifying IMPC patients according to T and N stages, the OS of T2-stage patients who received BCT was better than that of patients who received mastectomy, and there was no statistical difference in other stages. Moreover, the BCSS of IMPC patients treated with BCT had no significant difference after stratifying for T and N stages (**Figures 3**, **4**).

### Impact of Various Factors on Survival and Stratified Analysis of Overall Survival

Univariate Cox regression model analysis showed that older age (≥65 years old), single status, large-size tumor, lymph node metastasis, and mastectomy contributed to lower OS, while Asian or Pacific Islander and American Indian/Alaska Native race, ER positive, PR positive, and radiation therapy were associated with higher OS (**Table 2**). In addition, single-status patients, higher tumor grade (III), larger tumor size (>5 cm), lymph node metastasis, and treatment with a mastectomy had lower BCSS (**Table 3**); however, positive ER and PR and radiation therapy were protective factors for BCSS. Furthermore, univariate Cox regression model analysis showed that patients who received BCT had superior OS and BCSS compared with those who received mastectomy (HR = 1.590, 95% CI: 1.074–2.356, *p* = 0.021; HR = 2.395, 95% CI: 1.314–4.364, *p* = 0.004; respectively). In multivariate Cox regression model analysis, older age (≥65 years old) and radiation therapy were independent risk factors for OS (HR = 2.490, 95% CI: 1.343–4.615, *p* = 0.004; HR = 0.512, 95% CI: 0.280–0.938, *p* = 0.030; respectively) but not for BCSS (HR = 1.907, 95% CI: 0.833–4.364, *p* = 0.126; HR = 0.511, 95% CI: 0.237–1.099, *p* = 0.086; respectively). Single status and PR positive were independent risk factors for OS and BCSS. Interestingly, patients with N3 stage had worse OS (HR = 2.856, 95% CI: 1.361–5.995, *p* = 0.006) compared with those with N0 stage, while patients with N1 and N3 stages had worse BCSS compared with those with N0 stage (HR = 2.223, 95% CI: 1.026–4.817, *p* = 0.043; HR = 3.749, 95% CI: 1.337–10.510, *p* = 0.012; respectively). Radiotherapy was a protective factor for OS but not for BCSS (HR = 0.512, 95% CI: 0.280–0.938, *p* = 0.030; HR = 0.511, 95% CI: 0.237–1.099, *p* = 0.086; respectively). No difference in OS and BCSS was found between the BCT and mastectomy groups (HR = 0.727, 95% CI: 0.369–1.432, *p* = 0.357; HR = 0.762, 95% CI: 0.302–1.923, *p* = 0.565; respectively). We further performed a stratified analysis based on T stage. As shown in **Table 4**, in the multivariate analysis stratified by T stage, the OS of the BCT group for T2 stage was better than the mastectomy group (HR = 0.333, 95% CI: 0.149–0.741, *p* = 0.007), and no significant difference in OS was found between the BCT and mastectomy groups for patients with T1 and T3 disease (HR = 1.116, 95% CI: 0.608–2.050, *p* = 0.722; HR = 3.328, 95% CI: 0.693–15.974, *p* = 0.133, respectively).

**Table 2:** Univariate and multivariate analysis of OS of patients with early-stage IMPC.

Characteristics	OS			
	Univariate analysis		Multivariate snalysis	
	HR (95% CI)	*p*-value	HR (95% CI)	*p*-value
**Age (years)**				
<50	Reference	–	Reference	–
50–64	1.063 (0.586–1.929)	0.841	1.076 (0.581–1.992)	0.816
65–80	2.003 (1.143–3.510)	0.015	2.490 (1.343–4.615)	0.004
>80	4.258 (2.122–8.547)	<0.001	3.723 (1.702–8.143)	0.001
**Race**				
White	Reference	–	Reference	–
Black	1.471 (0.884–2.449)	0.137	1.126 (0.664–1.911)	0.659
Others	0.354 (0.130–0.965)	0.043	0.361 (0.131–1.000)	0.050
**Marital status**				
Married	Reference	–	Reference	–
Single	2.378 (1.606–3.522)	<0.001	2.006 (1.321–3.047)	0.001
**Grade**				
I	Reference	–		
II	0.960 (0.471–1.960)	0.912		
III	1.338 (0.654–2.740)	0.426		
IV	1.836 (0.565–5.966)	0.312		
**T stage**				
T1	Reference	–	Reference	–
T2	1.556 (1.014–2.388)	0.043	1.549 (0.980–2.449)	0.061
T3	3.319 (1.982–5.556)	<0.001	3.581 (1.758–7.295)	<0.001
**N stage**				
N0	Reference	–	Reference	–
N1	1.148 (0.728–1.809)	0.553	1.514 (0.923–2.484)	0.101
N2	1.388 (0.744–2.591)	0.303	1.085 (0.496–2.375)	0.838
N3	2.624 (1.507–4.570)	0.001	2.856 (1.361–5.995)	0.006
**ER status**				
Negative	Reference	–	Reference	–
Positive	0.389 (0.248–0.610)	<0.001	0.414 (0.211–0.810)	0.010
**PR status**				
Negative	Reference	–	Reference	–
Positive	0.500 (0.337–0.740)	0.001	0.644 (0.362–1.146)	0.135
**Surgery**				
BCT	Reference	–	Reference	–
Mastectomy	1.590 (1.074–2.356)	0.021	0.727 (0.369–1.432)	0.357
**Radiation**				
No	Reference	–	Reference	–
Yes	0.611 (0.417–0.897)	0.012	0.512 (0.280–0.938)	0.030
**Chemotherapy**				
No	Reference	–	Reference	–
Yes	0.736 (0.503–1.079)	0.116	0.630 (0.390–1.018)	0.059

OS, overall survival; HR, hazard ratio; 95% CI, 95% confidence interval; BCT, breast-conserving therapy.

**Table 3:** Univariate and multivariate analyses of BCSS of patients with early-stage IMPC.

Characteristics	BCSS			
	Univariate analysis		Multivariate analysis	
	HR (95% CI)	*p*-value	HR (95% CI)	*p*-value
**Age (years)**				
<50	Reference	–	Reference	–
50–64	0.857 (0.409–1.796)	0.684	0.927 (0.422–2.038)	0.851
65–80	1.096 (0.522–2.300)	0.808	1.907 (0.833–4.364)	0.126
>80	2.385 (0.893–6.373)	0.083	2.921 (0.933–9.149)	0.066
**Race**				
White	Reference	–	Reference	–
Black	1.129 (0.508–2.511)	0.766	0.661 (0.284–1.535)	0.335
Others	0.339 (0.082–1.400)	0.135	0.367 (0.085–1.589)	0.180
**Marital status**				
Married	Reference	–	Reference	–
Single	3.248 (1.802–5.853)	<0.001	3.524 (1.843–6.738)	<0.001
**Grade**				
I	Reference	–	Reference	–
II	3.075 (0.408–23.154)	0.276	2.936 (0.378–22.797)	0.303
III	8.845 (1.208–64.771)	0.032	5.305 (0.692–40.684)	0.108
IV	4.274 (0.267–64.401)	0.305	3.182 (0.180–56.346)	0.430
**T stage**				
T1	Reference	–	Reference	–
T2	1.815 (0.952–3.462)	0.070	1.336 (0.665–2.885)	0.416
T3	5.827 (2.957–11.485)	<0.001	3.696 (1.412–9.675)	0.008
**N stage**				
N0	Reference	–	Reference	–
N1	1.922 (0.942–3.923)	0.073	2.223 (1.026–4.817)	0.043
N2	3.090 (1.320–7.235)	0.009	1.355 (0.449–4.093)	0.590
N3	5.515 (2.515–12.094)	<0.001	3.749 (1.337–10.510)	0.012
**ER status**				
Negative	Reference	–	Reference	–
Positive	0.225 (0.127–0.398)	<0.001	0.349 (0.137–0.887)	0.027
**PR status**				
Negative	Reference	–	Reference	–
Positive	0.319 (0.185–0.551)	<0.001	0.557 (0.235–1.317)	0.182
**Surgery**				
BCT	Reference	–	Reference	–
Mastectomy	2.395 (1.314–4.364)	0.004	0.762 (0.302–1.923)	0.565
**Radiation**				
No	Reference	–	Reference	–
Yes	0.502 (0.292–0.866)	0.013	0.511 (0.237–1.099)	0.086
**Chemotherapy**				
No	Reference	–	Reference	–
Yes	1.503 (0.848–2.664)	0.163	0.979 (0.487–1.969)	0.952

BCSS, breast cancer-specific survival; HR, hazard ratio; 95% CI, 95% confidence interval; BCT, breast-conserving therapy.

**Table 4:** Univariate and multivariate regression analysis stratified according to T stage.

T stage	OS			
	Univariate analysis		Multivariate analysis	
	HR (95% CI)	*p*-value	HR (95% CI)	*p*-value
T1 BCT *vs.* mastectomy	1.175 (0.648–2.131)	0.596	1.116 (0.608–2.050)	0.722
T2 BCT *vs.* mastectomy	0.418 (0.191–0.914)	0.029	0.333 (0.149–0.741)	0.007
T3 BCT *vs.* mastectomy	1.798 (0.411–7.871)	0.436	3.328 (0.693–15.974)	0.133

OS, overall survival; HR, hazard ratio; 95% CI, 95% confidence interval; BCT, breast-conserving therapy.

## Discussion

Our results showed that the long-term survival advantage of women with early IMPC receiving BCT is equivalent to those undergoing mastectomy. Interestingly, the OS of patients with T2 stage receiving BCT was better than patients undergoing mastectomy. Our observations were based on data collected from 1,203 women in the SEER database and suggested that when BCT and mastectomy are feasible for early-stage IMPC treatment, BCT should be recommended, especially for patients with T2 stage.

IMPC is a rare histological subtype that predominantly affects women over 50 years old and is associated with a poor prognosis due to its invasiveness (3). The 5-year overall survival rates have been reported to range from 63% to 82.9% (18, 19). Histologically, IMPC consists of small clusters of tumor cells lying within clear stromal spaces which resemble dilated vascular channels. Immunohistochemically, IMPC exhibits an “inside-out” pattern of EMA expression (5, 7, 19). There is no significant difference in imaging findings between IMPC and invasive ductal carcinoma (IDC) as both present as high-density lesions on mammography and enhance significantly with MRI (20). Since most IMPC patients belong to the luminal biological subtype (21), chemotherapy has usually been ineffective in IMPC patients (22). Compared with IDC, IMPC has a greater potential risk of LRR. Therefore, radiotherapy may play an important role in improving LRR of patients with IMPC. A study reported that in patients that did not undergo radiotherapy, the incidence of LRR was significantly higher in the IMPC group than in the IDC group (19). Accordingly, many researchers recommended that patients with IMPC should receive radiotherapy (20, 23). Meng et al. (24) demonstrated that radiation therapy was significantly associated with improved LRR after mastectomy in IMPC. However, the clinical value of BCT for IMPC is still unclear.

In recent years, the optimal extent of breast resection for breast cancer has transitioned from wide resection to narrow surgical resection margins. Halsted’s radical mastectomy for breast cancer, which involved removing the whole breast, pectoralis major muscle, pectoralis minor muscle, and axillary lymph nodes, gradually became the standard treatment for breast cancer (25). However, subsequent studies showed that extensive resection did not improve survival rates. Therefore, in recent years, narrowing the extent of breast resection and postoperative adjuvant therapy has been advocated to improve patient survival. BCT which consists of lumpectomy and postoperative radiotherapy has become a standard of care in localized breast cancer (26). During lumpectomy in BCT, the tumor is removed, and the normal breast shape is maintained with minimal tissue damage. Studies have shown that patients that underwent BCT exhibited better physical and mental health and better quality of life (27).

The survival benefits brought by BCT warranted further investigations. Interestingly, randomized clinical trials showed similar survival rates in primary breast cancer patients who received BCT and mastectomy (8–10). Over the past few decades, with increased and early-stage breast cancer screening, the survival rate of breast cancer patients has significantly improved with multiple treatment modalities available, including chemotherapy, radiotherapy, endocrine therapy, and targeted therapy. Large-scale cohort studies have shown higher survival rates in patients with early-stage breast cancer with BCT than with mastectomy. For instance, Hartmann-Johnsen et al. (13) reported better OS and BCSS in women with early-stage breast cancer treated with BCT. The authors emphasized that differences in tumor biology and adjuvant systemic therapy could not fully explain this benefit. Furthermore, a Canadian study (14) on women with locally advanced breast cancer showed better patient outcomes with BCT. Importantly, Mirelle et al. (15) advocated that BCT should be the first choice for most breast cancer patients when both treatments are applicable. In 2016, Marissa et al. (16) controversially reported that BCT improved 10-year OS compared with mastectomy after hierarchical analysis of disease stages and adjustment of confounding variables. Similarly, BCT had a higher overall survival rate than mastectomy for breast cancer in a propensity score matching study based on the SEER database by Wrubel et al. (28). However, most of these studies included invasive ductal breast cancer (IDC) patients. Since IMPC is more prone to lymphatic invasion and higher lymph node metastasis, axillary lymph node dissection and extensive breast resection are recommended to obtain greater resection margins and lower recurrence rates (20, 23, 29). However, some studies found that this approach did not improve prognosis (6, 30). Survival analysis showed that IMPC patients in the BCT group had better OS and BCSS than those in the mastectomy group. In order to eliminate potential selection bias in BCT or mastectomy, further analysis was performed on IMPC patients after stratifying for T and N stages. We found that for breast cancer patients with T2 stage, the OS of the BCT group was better than the mastectomy group. In addition, after the inclusion of significant univariate variables, multivariate Cox regression model analysis showed that older age (≥65 years old), single, larger tumor (>5 cm), and lymph node positive (N3) were associated with poor OS, while ER-positive breast cancer and radiotherapy were associated with good OS. Our study also found that T and N stages were independent risk factors for BCSS in IMPC patients. Interestingly, during multivariate analysis, the OS or BCSS of patients receiving BCT did not improve significantly compared with those receiving mastectomy. After stratifying patients according to the T stage, better OS was found for patients with T2 disease in the BCT group than the mastectomy group, and no significant difference in OS was found between the BCT and mastectomy groups for T1 and T3 disease. According to the NCCN guidelines for breast cancer surgery, patients with tumor diameter less than 3 cm and stage III disease can consider breast-conserving surgery after preoperative chemotherapy (17). From clinical experience, patients with T2 stage disease should generally choose neoadjuvant chemotherapy before BCT; only patients with good responses to neoadjuvant chemotherapy should receive BCT. This approach leads to a better patient prognosis than those undergoing mastectomy. In conclusion, to the best of our knowledge, this is the first study to explore the impact of different surgical methods on the prognosis of early-stage IMPC patients. One main strength of this study was the long follow-up interval, and the large patient population studied, enabling us to provide real-world information on treatment approach selection for women diagnosed with breast invasive micropapillary carcinoma. Our study mainly focused on T1-3N0-3M0 patients, and most of these patients had the opportunity to choose BCT. Overall, our results showed that lumpectomy plus radiotherapy is an effective treatment strategy for invasive micropapillary breast carcinoma patients compared with mastectomy.

We also recognize several limitations of this study. First of all, although we tried to ensure the accuracy of the data; data obtained from the SEER database may have been subjected to selection bias and data input errors. Moreover, tumor-related information was unavailable, including multifocality or multicentricity, molecular typing and secondary surgery rates. However, it is widely acknowledged that patients with multifocal/multicentric tumors are not appropriate candidates for BCT. Therefore, adjusting for these variables was not feasible. Although it is unclear whether the two groups of patients received standard endocrine therapy, according to the positivity rates of ER and PR, we estimated the proportion of patients who underwent endocrine therapy since endocrine therapy is indicated in IMPC patients with positive ER and PR. Accordingly, we believe our findings are reliable. Moreover, no data on local recurrence and disease-free survival rates were available in the SEER database. Indeed, the primary outcome of this study was the survival rate of IMPC patients, which was influenced by other factors, including local recurrence and disease free. However, we could not determine the incidence of lymph node metastasis and tumor thrombus invasion in IMPC patients. Last, we did not analyze the entire IMPC patient population since T4 breast cancer and distant metastatic breast cancer are contraindications to BCT. In a nutshell, we confirmed that BCSS and OS were comparable between BCT and mastectomy in IMPC patients. Surprisingly, patients with T2 stage disease had better OS with BCT compared with mastectomy. IMPC is a special type of rare breast cancer that needs further studies with large sample sizes to investigate the optimal approach for surgical management.

## Conclusion

Overall, we demonstrated that prognosis of early-stage IMPC with breast-conserving treatment was at least equivalent to treatment with mastectomy. When both procedures are applicable, BCT should be recommended as the standard surgical treatment, especially for patients with T2 disease.

## Data Availability Statement

Publicly available datasets were analyzed in this study. These data can be found here: https://seer.cancer.gov/data/.

## Ethics Statement

In accordance with national legislative and institutional requirements, this study does not require written informed consent to participate.

## Author Contributions

SW was responsible for the design of the project. YZ was responsible for the data analysis. All the authors participated in the writing of the final manuscript. All authors have read and approved the final submission.

## Funding

This study is supported by grants from China National Natural Science Youth Fund Committee (Grant No. 81902702), Shandong Province Clinical Key Specialist Project Construction Fund (20110731250), and the establishment and demonstration of regional collaborative-graded diagnosis and treatment service model and clinical path of domestic innovative digital diagnosis and treatment equipment (2018YFC0114705).

## Conflict of Interest

The authors declare that the research was conducted in the absence of any commercial or financial relationships that could be construed as a potential conflict of interest.

## Publisher’s Note

All claims expressed in this article are solely those of the authors and do not necessarily represent those of their affiliated organizations, or those of the publisher, the editors and the reviewers. Any product that may be evaluated in this article, or claim that may be made by its manufacturer, is not guaranteed or endorsed by the publisher.
