# Corrigendum: Survival Outcomes After Breast-Conserving Therapy Compared With Mastectomy for Patients With Early-Stage Invasive Micropapillary Carcinoma of the Breast: A SEER Population-Based Study

**DOI:** 10.3389/fonc.2022.890018

**Published:** 2022-04-13

**Authors:** Song Wang, Yiyuan Zhang, Fangxu Yin, Xiaohong Wang, Zhenlin Yang

**Affiliations:** ^1^ Department of Thyroid and Breast Surgery, Binzhou Medical University Hospital, Binzhou, China; ^2^ Department of Reproductive Endocrinology, Affiliated Reproductive Hospital of Shandong University, Jinan, China

**Keywords:** invasive micropapillary carcinoma, SEER, mastectomy, BCT, survival

In the published article, there was an error in affiliation 1. Instead of “Department of Thyroid and Breast Surgery, Affiliated Hospital of Binzhou Medical University, Binzhou, China”, it should be “Department of Thyroid and Breast Surgery, Binzhou Medical University Hospital, Binzhou, China”.

The authors apologize for this error and state that this does not change the scientific conclusions of the article in any way. The original article has been updated.

## Publisher’s Note

All claims expressed in this article are solely those of the authors and do not necessarily represent those of their affiliated organizations, or those of the publisher, the editors and the reviewers. Any product that may be evaluated in this article, or claim that may be made by its manufacturer, is not guaranteed or endorsed by the publisher.

